# Accuracy of estimates of serving size using digitally displayed food photographs among Japanese adults

**DOI:** 10.1017/jns.2022.102

**Published:** 2022-11-24

**Authors:** Nana Shinozaki, Kentaro Murakami

**Affiliations:** Department of Social and Preventive Epidemiology, School of Public Health, The University of Tokyo, 7-3-1 Hongo, Bunkyo-ku, Tokyo 113-0033, Japan

**Keywords:** Food photograph, Japan, Serving size, Validation study

## Abstract

We evaluated the accuracy of the estimated serving size using digital photographs in a newly developed food atlas. From 209 food items in the food atlas, we selected 14 items with various appearances for evaluation. At the study site, fifty-four participants aged 18–33 years were served fourteen foods in the amount they usually ate. After they left, each food item was weighed by a researcher. The following day, the participants estimated the quantity of each food they served based on food photographs using a web-based questionnaire. We compared the weights of the foods the participants served (true serving sizes) and those determined based on the photographs (estimated serving sizes). For ten of the fourteen food items, significant differences were observed between the estimated and true serving sizes, ranging from a 29⋅8 % underestimation (curry sauce) to a 34⋅0 % overestimation (margarine). On average, the relative difference was 8⋅8 %. Overall, 51⋅6 % of the participants were within ±25 % of the true serving size, 81⋅9 % were within ±50 % and 93⋅4 % were within ±75 %. Bland–Altman plots showed wide limits of agreement and increased variances with larger serving sizes for most food items. Overall, no association was found between estimation errors and participant characteristics. The food atlas has shown potential for assessment of portion size estimation. Further development, refinement and testing are needed to improve the usefulness of the digital food photographic atlas as a portion size estimation aid.

## Introduction

One of the major sources of error in dietary assessment is the estimation of the food portion size^(1,2)^. Although weighing foods is considered accurate in quantifying the amount of food consumed, it is time-consuming, requires a high level of cooperation from respondents and may alter respondents’ eating behaviour^(3)^. Therefore, various portion size estimation aids have been developed as alternatives for assessing food portions, such as household measures, food models and food photographs^(1)^. Food photographs are less burdensome for respondents, easy to use for interviewees, portable and inexpensive, and cover a broad range of foods^(1,4–7)^. During data collection using food photographs, participants are asked to indicate the quantity of food they consumed by selecting a single picture or reporting a fraction, multiple or percentage of the amount shown in one photograph^(8)^. Today, food photographs with multiple-portion images are widely used in various dietary assessment methods, including automated self-administered 24-h recalls^(9–11)^.

Food photographs for portion size estimation should be designed for each country based on food availability, preferences and dietary intake data^(4,5)^. In addition, the characteristics of food photographs (e.g. the method of food presentation) may affect the perception, conceptualisation and memory of respondents, ultimately influencing the validity of the photographs^(12,13)^. Furthermore, it has been reported that the characteristics of respondents, such as age, affect the accuracy of portion size estimation^(1,2)^. Therefore, it is necessary to validate food photographs before using them as a portion size estimation aid in dietary surveys. To date, food photographs have been validated in various countries^(4,5,8,10,14–45)^. Although there are food photographs for estimationg portion size in Japan^(46)^, these were developed and validated food photographs in an elderly population (≥60 years) consisting of mostly women (90 %) living in one prefecture in Japan. Moreover, the portion sizes of the images were based on general recipes rather than dietary data. Thus, the comprehensiveness and representativeness of the food photographs are questionable. Given that Japanese cuisines consist of various amorphous foods, portion size estimation aids using digital photographs of foods consumed in Japan should be carefully developed and validated^(43,47)^.

We recently developed a digital food photographic atlas to help estimate portion size in dietary surveys in Japan^(48)^. Briefly, it was developed based on dietary record (DR) data among 644 Japanese adults for 4–16 d (5512 d in total), including weekdays (working days) and weekend days (non-working days). From the 1962 food and dish items identified from the DR, we selected approximately 300 top items in terms of the frequency of consumption, the sum of the consumed amount and energy contribution in the entire population. After eliminating food and dish items that did not require a photograph for portion size estimation, 209 commonly consumed food and dish items were included in the food atlas. Of these, 105 items are presented as a series of three to seven photographs showing gradually increasing portion sizes, while 104 items are shown as guide photographs representing a range of portion sizes and food varieties in one photograph. Portion sizes were determined based on market research and the distribution of food consumption in the DR. Moreover, the food atlas includes photographs of thirty-four household measurement items, such as cups and glasses.

Although the food atlas can be used as a portion size estimation tool in a self-administered 24-h dietary recall in the future, its validity has not been evaluated. Therefore, the present study aimed to evaluate the validity of images from the newly developed food atlas as a portion size estimation aid for Japanese adults. We evaluated the accuracy of the estimated serving size (the amount of food as served) rather than portion size (the amount of food as consumed). This was due to difficulties in asking study participants to eat a test meal during the infection control of coronavirus disease 2019 (COVID-19). In addition, we explored the association between estimation errors and participant characteristics, such as sex and age.

## Methods

### Selection of food items

Since it was infeasible to assess the validity of all food items in the food atlas^(48)^ with limited research resources, this study evaluated representative food items. Based on the number of food items (*n* 9–13) assessed in previous validation studies on food photographs^(29,34,49,50)^, we decided to evaluate approximately ten foods which could include at least one item from different food categories: amorphous/soft foods, liquid, spread, single-unit foods and small pieces^(29)^. A set of representative food items was selected considering various appearances, consistencies and textures^(4,6,10)^, mainly containing ingredients from different food groups^(34)^. We selected curry and rice, salad and white rice as amorphous/soft foods; dressing, coffee and miso soup as a liquid; margarine as spread; grilled mackerel and bananas as single-unit foods; and cookies, Japanese fried chicken and simmered squash as foods in small-piece shapes. The description and portion size of these twelve food items are listed in Table 1.

**Table 1.** Description of foods and food photographs selected for this study

Food characteristic	Food	Type of photograph		Number of portion sizes	Weight of each portion in photograph (g)
		Series*	Guide^†^		
Amorphous/soft	Curry and rice (Japanese curry made with carrot, potato and beef served with white rice)	✓		7	160⋅7, 208⋅7, 272⋅7, 351⋅7, 456⋅7, 593⋅7, 770⋅7
	Salad (mixed salad with corn, cabbage, tomato and cucumber)	✓		7	27⋅2, 39⋅2, 56⋅4, 81⋅2, 116⋅8, 168⋅2, 242⋅0
	A bowl of white rice	✓		Six for small and medium bowls; and seven for a large bowl	59⋅8, 78⋅1, 102⋅4, 133⋅4, 173⋅8, 226⋅8 for small and medium bowls; 59⋅8, 78⋅1, 102⋅4, 133⋅4, 173⋅8, 226⋅8, 295⋅8 for a large bowl
Liquid	Dressing (soy sauce-based dressing with chopped onion served on a small plate)	✓		7	3⋅0, 4⋅4, 6⋅2, 9⋅1, 13⋅0, 18⋅7, 27⋅0
	Coffee	✓		Seven for each of six cups	20–390 (increment per potion is 10–60 ml, depending on the cup)
	Miso soup (traditional Japanese soup made of miso paste, broth, green onion and tofu)	✓		Three for a small bowl; six for a medium bowl and seven for a large bowl	111⋅3, 133⋅2, 160⋅0 for a small bowl; 111⋅3, 133⋅2, 160⋅0, 192⋅0, 230⋅5, 276⋅5 for a medium bowl; 111⋅3, 133⋅2, 160⋅0, 192⋅0, 230⋅5, 276⋅5, 332⋅0 for a large bowl
Spread	Margarine	✓		7	2⋅0, 2⋅9, 4⋅3, 6⋅3, 9⋅3, 13⋅6, 20⋅0
Single-unit	Grilled mackerel (salted grilled mackerel, cut into bite-size pieces)	✓		7	19⋅7, 28⋅4, 41⋅0, 59⋅0, 82⋅0, 121⋅9, 164⋅5
	Bananas		✓	3	97⋅3, 146⋅6, 191⋅4
Small pieces	Cookies		✓	19	2⋅5, 2⋅7, 3⋅0, 3⋅2, 4⋅5, 4⋅9, 5⋅4, 5⋅4, 6⋅1, 6⋅2, 8⋅4, 8⋅8, 8⋅8, 11⋅2, 13⋅4, 15⋅0, 15⋅0, 20⋅0, 57⋅3
	Japanese fried chicken (bite-size deep-fried chicken pieces)		✓	5	12⋅1, 15⋅3, 21⋅3, 34⋅1, 60⋅9
	Simmered squash (simmered squash cut into small pieces)	✓		7	22⋅0, 35⋅0, 53⋅0, 83⋅0, 132⋅2, 209⋅4, 329⋅5

* A series of photographs show gradually increasing portion sizes.

† A guide photograph shows the range of portion sizes and food varieties in one photograph.

### Study participants

The study participants included only students and staff at the University of Tokyo due to the entrance restriction to a study site on campus during the COVID-19 pandemic. Recruitment was conducted through e-mail and social media (LINE and Twitter), which included a web link to a study website. Potential participants were encouraged to read the details of the study on the website, with the purpose of the study concealed^(51)^. The inclusion criteria were healthy students or faculty members of the University of Tokyo aged 18–65 years who could complete a web-based questionnaire using a computer. The exclusion criteria were individuals who had majored in nutritional science or cooking (e.g. dietitians and chefs), pregnant or lactating women, and those with severe visual or neurological deficits or severe food allergies. In addition, individuals who did not usually eat any of the three test foods (white rice, bread or bananas) were excluded. This was because, while this study asked the participants to serve test foods, if they had never or rarely eaten a certain test food, they were asked to imagine a similar food while serving the food, which was considered difficult for the three foods listed above due to their unique shape and characteristics. To check eligibility, a questionnaire was administered on the website, and those who met all these criteria could be applied for the study using an online reservation form. The numbers of male and female participants were controlled to be almost equal by controlling the available slots by sex. We aimed to include fifty participants based on the guideline of validation studies of food photographs^(51)^. Assuming a 20 % dropout rate^(4)^, sixty-three adults (thirty-three males and thirty females) were invited to participate in this study.

The study was conducted according to the guidelines laid down in the Declaration of Helsinki and all procedures were approved by the Ethics Committee of the Faculty of Medicine of the University of Tokyo (2021121NI-(1); approved 15 September 2021). Online informed consent was obtained from each participant by clicking the ‘I agree’ box on the online booking form. In addition, written informed consent was obtained from all the participants during their visit to the study site. Each participant received an Amazon gift card worth 2000 Japanese yen (approximately £12⋅5) when they completed the study.

### Study design

**Fig. 1.**
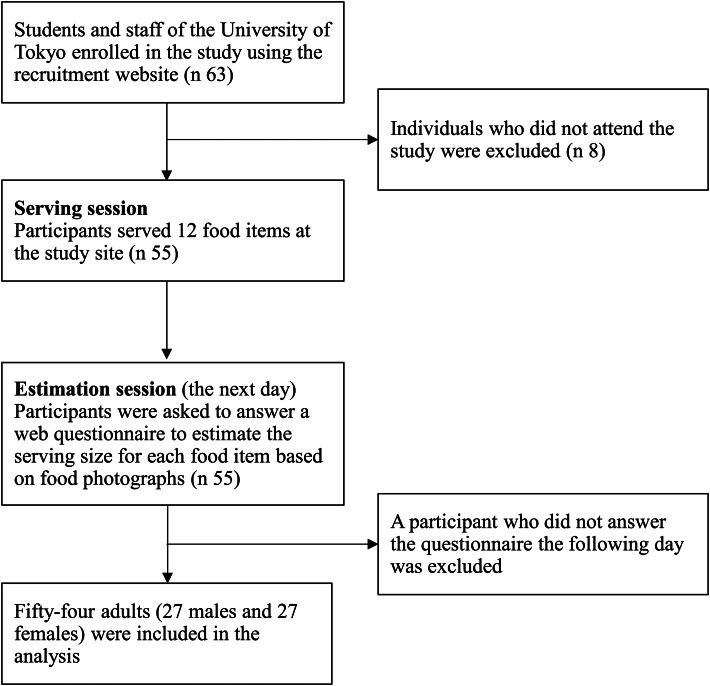
Flow diagram of the study.

Data were collected in November 2021. On the first day of the study, participants served test foods on plates at the study site (serving session). The next day, participants were asked about the type and amount of food they served using food photographs through a web-based questionnaire (estimation session). Thus, the study consisted of two separate sessions over 2 d for each participant (Fig. 1), which can replicate a situation similar to self-administered 24-h recalls. Prior to data collection, a pilot study was conducted with five students and staff in our department to improve the study protocol.

### Preparation of test foods and the study site for the serving session

Prior to the serving session, the test foods were purchased from local supermarkets and convenience stores. We bought several products of different shapes and sizes for bananas (*n* 5), cookies (*n* 6) and Japanese fried chicken (*n* 3). For other foods, one product was purchased for each item. The products purchased were identical to those shown in the photographs for several items (curry, white rice, dressing, margarine, simmered squash and one of the three types of Japanese fried chicken). In contrast, for other food items, the products purchased were different from those shown in the photographs.

Each food was prepared on an appropriate cooking utensil, such as rice cookers, pots and plates. The amount of food prepared was at least 1⋅5 times greater than the seventh portion size in the food atlas^(48)^, except for some cookies with a large bulk per unit weight, which were prepared in quantities appropriate for the plate. All foods were arranged on tables at a study site, which was prepared to be similar to the situation of serving food at home (Supplementary Fig. S1(a)). The twelve test foods were categorised into lunch, snack, dinner and other menus, considering the standard number and combinations of dishes at meals in the Japanese diet^(52)^. The lunch menu consisted of curry and rice, salad, dressing and bananas; the snack menu comprised cookies and coffee; the dinner menu comprised a bowl of white rice, miso soup, grilled mackerel (or Japanese fried chicken) and simmered squash; and the other menu was margarine, accompanied by a slice of bread. Each menu was separately placed on the table (Supplementary Fig. S1(b)–(e)). A total of sixty-four types of various tableware (plates, cups, rice bowls, soup bowls, chopsticks, spoons, knives and forks), including twenty used in the photos of the test foods, were also prepared on a separate table (Supplementary Fig. S1(f)). Each tableware was assigned an identification (ID), weighed on a calibrated cooking scale (KW-320, Tanita, Tokyo, Japan) and measured up to 300 g in 0⋅1 g, 300–1500 g in 0⋅5 g and 1500–3000 g in 1 g.

### Serving session

Each serving session was conducted by one person at a time (seven participants per day). Before each session, the author (K. M.) weighed all test foods using a calibrated cooking scale. The participants received verbal and written explanations of the study protocol from the author (N. S.) in a space separated from the study site. We told the participants in advance that they would not eat the food to prevent the expectation of eating the food from altering their behaviours^(18)^. The participants were requested to answer a paper questionnaire asking, ‘How hungry are you right now?’. Potential responses were provided on a 5-point Likert scale ranging from ‘extremely hungry’ to ‘not hungry’. The questionnaire also asked whether the participants ate each test food. If there was a food that had never or rarely been eaten, the participants were instructed to serve the food imagining similar shapes of the food (e.g. peanut butter instead of margarine)^(18)^ to prevent missing information about the serving size of the food. This procedure also imitates an actual self-administered 24-h dietary recall method in which some food items are estimated using portion images that closely resemble those foods^(53)^.

Participants were then invited to the study site. First, the participants were told, ‘Assuming this menu is your lunch, please serve each food on a plate in the amount you usually eat’. They were asked to use tableware (e.g. plates, rice bowls and cups) similar to those they usually used at home. In addition, they were asked to select the appropriate cutlery (i.e. chopsticks, spoons, knives and forks) for the meal. After serving the food, the participants were asked to sit in front of the table and arrange each plate and cutlery on a tray; thus, their eye level would be closer to the real eating situation. This process was repeated in the order of snack, dinner and margarine. For bananas, participants were asked to choose one of the available variations. For cookies and Japanese fried chicken, the participants were first asked to choose one of the variations available and then serve it in the amount they would eat. For the dinner menu, participants were asked to serve white rice, miso soup, simmered squash and grilled mackerel as one menu. They were then asked to serve Japanese fried chicken instead of grilled mackerel, with the other items remaining.

After each participant left the room, the author (K. M.) weighed the plate or cup in which each food item was served. The author (N. S.) recorded the total weight of the plate and food and the IDs of the plates and cutlery on measurement record sheets. The time taken to complete the process of serving food was also recorded. The food served was returned to each cooking utensil, if possible. Moreover, the food was refilled as needed.

### Estimation session

On the morning of the next day, we sent each participant an e-mail containing a link to a web-based questionnaire asking him/her to estimate the amount of each food served on the previous day based on the food photographs. The participants were asked to answer the questionnaire within 12 h after the reception of the e-mail to replicate the situation of 24-h recalls.

**Fig. 2.**
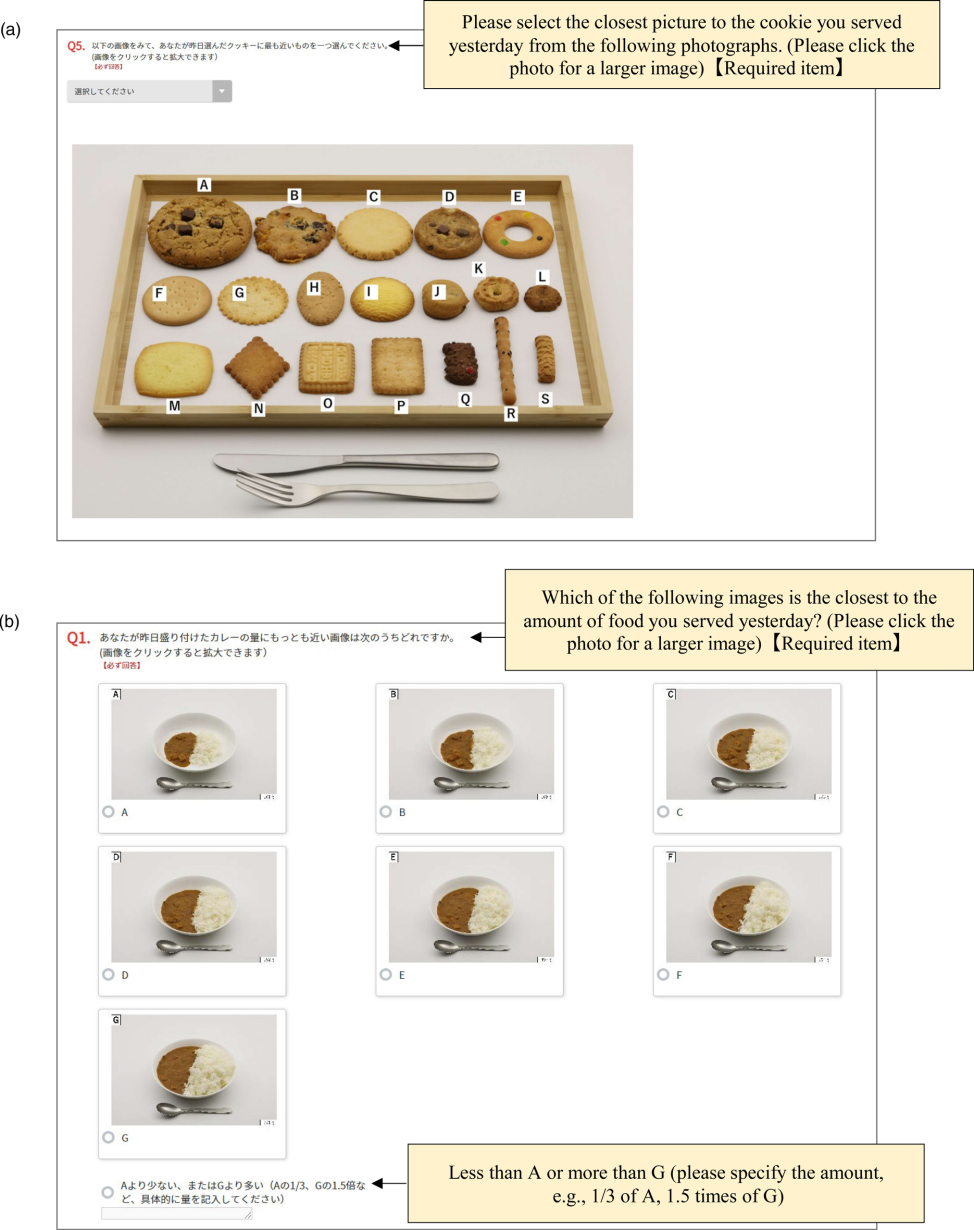
Example screens from the web-based questionnaire, with English translation shown in the boxes. (a) Screenshot of the question to ask the type of cookies using a guide photograph and (b) screenshot of the question to ask the serving size of curry and rice using a series of photographs.

The serving size of each food was determined using either a guide photograph (Fig. 2(a)) or a series of photographs (Fig. 2(b)). Both types of photographs contained one or two standard reference objects (e.g. knife and fork) to help estimate the quantity of food and dishes and the size of the plate^(54,55)^. The guide photographs were used for bananas, cookies and Japanese fried chicken, with one photograph representing various sizes and types of food (Supplementary Fig. S2(i), (j), and (k), respectively). The number of variations shown in the photographs was three for bananas, nineteen for cookies and five for Japanese fried chicken (Table 1). For these items, the participants were first asked to select one that looked similar to the food they had served on the previous day (Fig. 2(a)). For cookies and Japanese fried chicken, the participants further answered the number of pieces they served.

A series of photographs, which show gradually increasing portion sizes (Table 1), were used for the other items (examples of photographs shown in Supplementary Fig. S2 (a) for curry and rice, (b) for salad, (c) for white rice, (d) for dressing, (e) for coffee, (f) for miso soup, (g) for margarine, (h) for grilled mackerel and (l) for simmered squash). Since curry and rice are usually served on one plate and eaten together in Japan, the photos also showed their portions on a single plate, from which the participants were asked to choose one (Fig. 2(b)). The participants were asked to choose the image closest to the amount of food they had served on the previous day (Fig. 2(b)). There were seven portion sizes, except for white rice and miso soup on small and medium bowls (3–6 portions). Moreover, the participants could report the serving size other than the specific photograph in a free-text field, such as less than the smallest, more than the largest or between pictures. The serving sizes of white rice, miso soup and coffee were asked through a two-step question. First, the participants were shown photographs of different rice bowls (*n* 3), soup bowls (*n* 3) and coffee cups (*n* 6), and were asked to choose one that was similar to the tableware they used in the serving session (Supplementary Fig. S2(c), (f), and (e), respectively). Next, the participants were shown images of portions for each tableware, from which they were asked to choose one.

The questionnaire also included questions on sex, age (years), body height (cm), weight (kg), occupation (undergraduate students, graduate students or faculty members) and cooking frequency per week (less than once, two or three, four or five or more than six). To prevent any missing responses, the questionnaire was designed such that the participants could not move to the next page before answering all questions.

To unify the layout of the answer screen as consistently as possible among participants, the participants were required to use a computer to answer the questionnaire and not use a smartphone or tablet. The participants answered the questionnaire at any location. The web-based questionnaire was developed using the online survey platform Questant (Macromill, Inc., Tokyo, Japan).

### Calculation of the true and estimated serving sizes

During the serving session, the amount of food served (true serving size) was calculated for each food item by subtracting the pre-weighed weight of each plate or cup from the total weight of the plate and food.

For the estimation session, the estimated serving sizes using the food photographs (estimated serving size) were computed based on the weight of the food in the selected photograph in the food atlas database^(48)^. The estimated serving sizes of the Japanese fried chicken and cookies were calculated as the weight of a single piece selected multiplied by the number of pieces reported. If the serving size was estimated as a multiple of a specific photograph (e.g. 0⋅9 times of photo A) in the free-text field, this value was used to calculate the serving size. If no specific value was given, the serving size was calculated by multiplying the food weight of the selected photograph by 0⋅8 for the answer ‘a little less’ or 1⋅2 for the answer ‘a little more’. These values were determined based on the average weight ratio between the food weights for each photo in the series of photographs (0⋅7 for decreasing portions and 1⋅4 for increasing portions). When the number between photos was reported, the mean value of the two food weights was used.

### Statistical analysis

For the analysis, curry and rice were analysed separately for the whole curry and rice, curry sauce and white rice, resulting in a total of fourteen items for evaluation. We assessed the difference between the true and estimated serving size as follows. First, the absolute difference (g) between the true and estimated serving sizes was evaluated using the paired *t*-test and Wilcoxon signed-rank test. The Wilcoxon signed-rank test results were presented because the two tests yielded similar results. Second, the relative difference (%) was calculated by subtracting the true serving size from the estimated serving size, divided by the true serving size and multiplied by 100. Moreover, the percentage of participants selecting serving sizes within ±10, ±25, ±50 and ±75 % of the true serving size was calculated. Furthermore, Bland–Altman plots^(56)^ were used to assess the degree of agreement between the true and estimated serving sizes. We also used linear regression analysis to examine the proportional bias between true and estimated serving sizes^(57)^.

The basic characteristics of the participants were presented as the mean and standard deviation (sd) or the number of participants (%). Body mass index (BMI) was calculated as body weight (kg) divided by the square of body height (m^2^). The cooking frequencies of ‘four or five’ and ‘more than six’ were combined because each category included a few participants. Similarly, the hunger level was grouped into three categories: ‘hungry’, ‘neither’ and ‘not hungry’.

We assessed the association between participant characteristics (sex, age, BMI, occupation, cooking frequency and hunger level) and errors in serving size estimation. Age and BMI were converted into categorical variables using the median, generating younger (18–23 years, *n* 31) or older groups (24–33 years, *n* 23), and lower BMI (16⋅8–20⋅6 kg/m^2^, *n* 27) or higher BMI groups (20⋅8–30⋅8 kg/m^2^, *n* 27), respectively. The occupation categories ‘graduate student’ and ‘faculty member’ were combined because the latter included a limited number of participants. To compare the mean relative difference among categories, the Wilcoxon rank-sum test and the Kruskal–Wallis test were used as appropriate.

All statistical analyses were performed using Statistical Analysis System (SAS) version 9.4 (SAS Institute Inc., Cary, NC, USA). Two-sided *P*-values < 0⋅05 were considered statistically significant.

## Results

### Participant characteristics

Of the sixty-three participants enrolled in the study, eight did not attend, and one did not answer the web-based questionnaire (Fig. 1). Consequently, fifty-four participants (twenty-seven men and twenty-seven women) aged 18–33 years were included in the analysis. Table 2 shows the participant characteristics. The mean age was 23⋅6 years (sd: 3⋅2), and the mean BMI was 20⋅8 kg/m^2^ (sd: 2⋅5). Approximately half (52 %) of the participants were undergraduate students. Most participants (69 %) reported habitual cooking more than twice a week. The mean duration of the experiment for serving food was 11⋅5 min (sd: 2⋅2). The median ratio of the participants who had never or rarely eaten the test food for each food item was 2⋅8 % (interquartile range: 0–8⋅3).

**Table 2.** Participant characteristics (*n* 54)

Variables	Values			
	Mean	sd	*n*	%
Sex				
Male			27	50
Female			27	50
Age (years)	23⋅6	3⋅2		
Body height (cm)	165⋅2	8⋅9		
Body weight (kg)	57⋅1	10⋅6		
Body mass index (kg/m^2^)	20⋅8	2⋅5		
Occupation				
Undergraduate student			28	52
Graduate student			25	46
Faculty member			1	2
Cooking frequency (days per week)				
Less than once			17	31
Two or three			22	41
Four or more			15	28
Hunger				
Hungry			26	48
Neither			7	13
Not hungry			21	39

sd, standard deviation.

### Differences between the true and the estimated serving sizes

Table 3 shows the differences between the true and estimated serving sizes for each food item. In ten of the fourteen food items examined, a significant difference was observed between estimated and true serving sizes. The mean estimated serving sizes of four items (curry and rice, curry sauce, cookies and a bowl of rice) were smaller than the true serving size, whereas those of six items (salad, banana, coffee, miso soup, Japanese fried chicken and margarine) were larger than the true serving size. The mean relative difference between the estimated and true serving sizes ranged from a 29⋅8 % underestimation for curry sauce to a 34⋅0 % overestimation for margarine. On average, the relative difference was 8⋅8 %.

**Table 3.** Difference between the true and the estimated serving sizes of food and drink items (*n* 54)

Meal type	Item	True serving size (g)		Estimated serving size (g)		Absolute difference* (g)			Relative difference^†^ (%)		Percentage of participants with a difference between true and estimated serving size within each percentage			
		Mean	sd	Mean	sd	Mean	sd	*P*^‡^	Mean	sd	±10 %	±25 %	±50 %	±75 %
Lunch	Curry and rice	405⋅7	115⋅5	336⋅6	132⋅3	−69⋅1	104⋅6	<0⋅0001	−16⋅2	23⋅0	27⋅8	66⋅7	90⋅7	100
	White rice	199⋅4	63⋅6	193⋅9	74⋅6	−5⋅4	58⋅6	0⋅47	−0⋅1	28⋅5	11⋅1	31⋅5	85⋅2	100
	Curry sauce	206⋅3	58⋅3	142⋅7	58⋅1	−63⋅6	53⋅9	<0⋅0001	−29⋅8	21⋅7	25⋅9	57⋅4	94⋅4	100
	Salad	68⋅7	17⋅8	85⋅8	28⋅6	17⋅1	31⋅2	0⋅0002	31⋅4	50⋅1	11⋅1	40⋅7	70⋅4	83⋅3
	Dressing	7⋅0	3⋅6	7⋅8	4⋅9	0⋅8	3⋅9	0⋅11	19⋅0	53⋅8	11⋅1	33⋅3	70⋅4	88⋅9
	Banana	148⋅2	39⋅8	159⋅7	32⋅0	11⋅5	36⋅4	0⋅004	11⋅8	25⋅7	33⋅3	70⋅4	88⋅9	98⋅1
Snack	Cookies	50⋅2	34⋅4	47⋅8	32⋅9	−2⋅4	19⋅1	0⋅01	3⋅5	49⋅2	27⋅8	66⋅7	77⋅8	87⋅0
	Coffee	180⋅3	59⋅9	223⋅0	78⋅1	42⋅7	53⋅5	<0⋅0001	27⋅0	34⋅3	35⋅2	55⋅6	77⋅8	90⋅7
Dinner	A bowl of rice	150⋅3	51⋅9	137⋅1	47⋅7	−13⋅2	44⋅6	0⋅02	−6⋅2	25⋅5	25⋅9	66⋅7	94⋅4	100
	Miso soup	163⋅5	44⋅4	196⋅4	50⋅4	32⋅9	56⋅5	0⋅0002	26⋅8	41⋅1	14⋅8	55⋅6	72⋅2	81⋅5
	Grilled mackerel	88⋅4	26⋅3	84⋅2	26⋅6	−4⋅2	27⋅2	0⋅14	−0⋅1	31⋅9	14⋅8	48⋅1	90⋅7	98⋅1
	Simmered squash	104⋅3	39⋅9	104⋅5	41⋅0	0⋅2	23⋅8	0⋅68	2⋅4	23⋅1	38⋅9	81⋅5	96⋅3	100
	Japanese fried chicken	155⋅6	47⋅6	187⋅3	93⋅3	31⋅7	71⋅0	0⋅002	19⋅5	43⋅4	7⋅4	18⋅5	64⋅8	96⋅3
Other	Margarine	4⋅9	2⋅9	5⋅8	3⋅8	0⋅9	2⋅6	0⋅005	34⋅0	74⋅2	24⋅1	29⋅6	72⋅2	83⋅3

sd, standard deviation.

* Absolute difference (g) = estimated serving size − true serving size. Thus, positive and negative values indicate overestimation and underestimation of the serving size, respectively.

† Relative difference (%) = ([estimated serving size − true serving size]/true serving size) × 100.

‡ Differences between the amounts served and estimated were tested using the Wilcoxon signed-rank test.

The lowest percentage of participants whose estimate was within ±10 % of the true serving size was observed for Japanese fried chicken (7⋅4 %), while the highest percentage was observed for simmered squash (37⋅0 %). On average, 51⋅6 % of the participants were within ±25 % of the true serving size, 81⋅9 % were within ±50 % and 93⋅4 % were within ±75 %.

### Agreement between the true and the estimated serving sizes

**Fig. 3.**
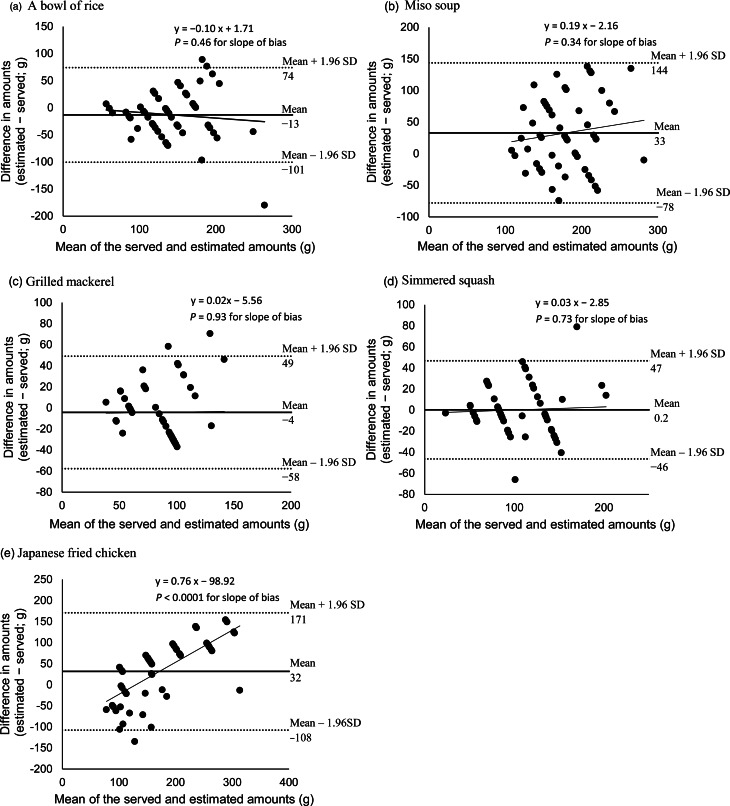
Bland–Altman plots assessing the agreement of the served and estimated food amounts in fifty-four Japanese adults: (a) a bowl of rice, (b) miso soup, (c) grilled mackerel, (d) simmered squash and (e) Japanese fried chicken. The solid line represents the mean difference, and the dotted line represents lower and upper limits of agreement with a solid regression line added.

The Bland–Altman plots are shown in Fig. 3 for four food items served for dinner (a bowl of rice, miso soup, grilled mackerel and simmered squash) and Supplementary Fig. S3 for other items. Because of the limited number of photographs in the portion size selection, most plots showed several diagonal rows of data points. The limits of agreement were wide for most food items, mainly because of increased dispersion with larger serving sizes. Linear regression analysis demonstrated that the slope of the mean bias for each food item was significantly different from 0 for five items (salad, dressing, coffee, Japanese fried chicken and margarine), indicating a proportional bias that differences between the true and estimated serving size increased as the mean food weight increased.

### Association between participant characteristics and estimation error

Overall, there were no relative differences in most food items among the categories of participant characteristics, including sex, age, BMI, occupation, cooking frequency and hunger level. However, the relative difference in the volume of coffee was larger in women than in men (mean relative difference: 33⋅3 % *v*. 20⋅8 %, *P* = 0⋅04). Moreover, compared with the younger group, the older group had a larger relative difference for miso soup (mean relative difference: 15⋅6 % *v*. 41⋅8 %, *P* = 0⋅02) and simmered pumpkin (mean relative difference: −6⋅4 % *v*. 14⋅2 %, *P* = 0⋅004). Furthermore, the estimated serving size of the simmered pumpkin was smaller than the true serving size among undergraduate students, whereas it was larger than that in the group of graduate students and university staff (mean relative difference: −5⋅0 % *v*. 10⋅3 %, *P* = 0⋅01).

## Discussion

### Main findings

To the best of our knowledge, this is the first study to assess the validity of comprehensive digital food photographs as a portion size estimation aid in Japan. The results showed significant differences between the estimated and true serving sizes for most food items, with wide limits of agreement. However, on average, the relative difference was small, and more than half of estimates were within ±25 % of the true serving size. These findings indicate that the food atlas can be used in portion size estimation. However, further development, refinement and testing are needed to improve the usefulness of the digital food photographic atlas as a portion size estimation aid.

### Comparison with previous studies

The acceptable accuracy of portion size measurement has not yet been established^(22,50)^. However, a previous study considered that the relative error within 25 % was acceptable^(5)^, and the present study showed a mean relative difference within this range (8⋅8 %). Similarly, previous studies, which asked adult participants to recall the consumed amount of food of the previous day, also showed a mean relative difference within the range: 3⋅6 % in the USA^(10)^, −4⋅1 % in Lebanon^(35)^ and 4⋅5 % in Nepal^(45)^.

In the present study, the relative difference between the true and estimated serving sizes was large among food items, indicating that the estimation accuracy varied across foods. Moreover, we observed both underestimation and overestimation of food serving sizes. These results are consistent with those of previous studies. For instance, the percentage errors across foods in studies using recall methods for adults ranged from −29 to +38 % in the UK^(17)^, −24 to 17 % in the USA^(10)^, −14 to +117 % in Belgium^(19)^, −7 to +9 % in Bolivia^(38)^, −25 to +6 % in Malawi^(36)^, −8 to 6 % in Burkina Faso^(39)^, −12 to +15 % in Lebanon^(35)^ and −8 to +82 % in the United Arab Emirates^(34)^. On average, in the present study, 51⋅6 % of the participants had estimates within ±25 % of the true serving size, which is higher than that of adults in the USA (38 %)^(10)^ and the Netherlands (35 %)^(50)^. Nevertheless, a direct comparison of the results is difficult because of the large differences in the study design, such as the target population, selection of food items and timing of estimation^(18)^.

The accuracy of portion size estimation is affected by the type, shape, texture and size of food^(1,7)^. Portion size estimation is particularly difficult for foods with certain characteristics, such as amorphous foods^(1,6,29,40)^. In contrast, foods consumed in a defined unit can be easily estimated. For instance, the proportion of reported portion sizes within 10 and 25 % of the true serving size was the largest for single-unit foods in previous studies^(10,50)^. However, we did not find a clear association between estimation errors and food characteristics. For example, although both grilled mackerel and bananas are single-unit foods, a non-significant difference between the true and estimated serving sizes was observed only for the grilled mackerel. Furthermore, the percentage of participants whose estimate was within ±10 % of the true serving size was lowest for Japanese fried chicken and highest for simmered squash, although these foods had small pieces in common. These results suggest that the accuracy of estimates may vary even among foods with similar characteristics, as previously reported^(39)^. Meanwhile, all foods depicted in the guide photographs (i.e. bananas, cookies and Japanese fried chicken) showed a significant difference between the estimated and true values, suggesting that the type of photograph may affect the accuracy of the estimates. However, a formal analysis comparing the estimation accuracy between food characteristics or types of photographs was hindered because of the small number of foods within each category.

The conditions of the study may also affect the estimation accuracy. The fact that participants had never or rarely eaten the test foods may have affected the accuracy of the estimates, whereas the percentage of such individuals was low for each food in this study. In addition, the percentages of subjects who estimated within 10 and 25 % of the true serving size were similar between foods for which the items prepared in the serving session were identical to those in the photographs (curry and rice, curry sauce, white rice, dressing, a bowl of rice, margarine and simmered squash) and those for the other foods (data not shown).

The limits of agreement were wide for most food items, indicating a large individual variation in the estimation ability. In general, estimation accuracy decreases as the portion size increases^(18,22)^. Indeed, we observed an increase in variance with increasing amounts for most foods, similar to previous studies^(21,34,39)^. Moreover, previous studies have indicated the presence of a ‘flat slope syndrome’, in which small portions are overestimated and large portions are underestimated^(4,5,8,36,40,58)^. For instance, a previous study reported that portions of ≥100 g were, on average, underestimated by 2⋅4 %, whereas small portions (<100 g) were overestimated by 17⋅1 %^(58)^. However, we observed that both the food items with the means of true serving sizes of ≥100 g (nine items) and <100 g (five items) were overestimated (mean relative difference, 3⋅9 and 17⋅6 %, respectively). Moreover, the proportional bias observed for several items (dressing, salad, coffee, Japanese fried chicken and margarine) indicated that the portion size is more likely to be overestimated as it increases. A previous study explained that one of the reasons for flat slope syndrome was that portion size options were limited to below the smallest portion and above the largest portion^(5)^. This could not have happened in this study since the participants were allowed to report a serving size smaller than the smallest food quantity and that larger than the largest one; nevertheless, no such answers were reported. As the number of participants and test foods was limited in this study, further studies on the association between portion size and estimation accuracy are required.

Previous studies have found inconsistent associations between errors in portion size estimation using food photographs and BMI^(12,22,34)^, sex^(4,17,19,22,34,40)^ and age^(4,22,34,43,59)^. Overall, the present study showed no difference in the errors in serving size estimation among the categories of participant characteristics. Therefore, the general error pattern associated with a particular food may be due to its photographic presentation rather than to specific participant characteristics^(17)^. In the present study, the serving size of coffee was overestimated in women than in men, which is inconsistent with a previous study^(19)^. Since there was a large variation in the study design in previous studies, further studies are needed to determine the effect of participant characteristics on the accuracy of portion size estimation.

### Strengths and limitations

The strength of the present study is its design, which imitates the situation of a 24-h dietary recall. It allowed evaluation of the accuracy of estimates concerning the conceptualisation of foods and memory while recalling the diet^(50)^. However, the present study had several limitations. First, the convenience sample consisted of young and highly educated individuals, which reduced the generalisability of the results. Moreover, participants may have been motivated to report the serving size accurately because they received a reward. Additionally, owing to limited research resources, the present study included the minimum number of participants recommended for validation studies of food photographs^(51)^. Consequently, our sample size may have resulted in non-significant results. A *post hoc* power analysis revealed that the four items that did not significantly differ between true and estimated serving sizes had inadequate statistical power, ranging from 11 % (simmered squash) to 42 % (dressing). Therefore, these findings should be interpreted with caution. The validity of food photographs should be evaluated in a larger population with diverse backgrounds, including children and older adults. Second, food photographs may have varied in resolution and size, depending on the devices used to answer the web questionnaire, possibly causing between-person variations in estimation accuracy. Previous studies have reported that the use of tablets showed less accuracy than the use of computer screen^(4)^, whereas the size of the images of food portions did not affect the accuracy of estimation in both children^(30,43)^ and adults^(29)^. Accordingly, we asked the participants to answer the web questionnaire using any computer available, and almost all participants (98⋅2 %) responded to the web questionnaire through the computer. Third, the food photographs were tested in a highly controlled setting. Although participants were not informed of the purpose of the study, they may have paid more attention to the amount of food. Moreover, they were asked to serve a limited number of foods that had almost the same appearance in the photographs, whereas people consumed a much wider variety of foods in the real world. The tableware variation in the serving session was also limited, including some of the same tableware as those in the photographs, which is unlikely to occur in a real-life situation. Therefore, our results should be considered a best-case scenario. Fourth, the number of food items validated in this study accounted for only 6 % of foods included in the food atlas^(48)^. However, because the test food items were selected to represent various food categories, appearances and food groups, the food atlas may be generally able to estimate portion sizes. Further validation studies with other photographs are needed to confirm the validity of the entire food atlas. Lastly, we could not evaluate the validity of the food photographs in estimating the portion size (the amount of food consumed) because the foods were not consumed for hygiene reasons. Previous studies in adults have suggested that food weights can be both underestimated and overestimated regardless of whether study participants ate^(10,17,38,45)^ or did not eat^(5,19,26,39)^ test foods. Meanwhile, studies on children have suggested that the amount of food consumed is estimated less accurately than the amount served because of the impact of errors in reporting leftovers^(14)^. Given the latter finding, the accuracy of food portion estimates may have been overestimated in this study. Therefore, for future development of a food atlas used in a 24-h recall method, the validity of food photographs should be assessed for actual food consumption using more photographs in a less controlled setting.

## Conclusion

We assessed the accuracy of the estimates of the amount of foods self-served by participants using digital food photographs from a recently developed food atlas. The results suggest that, for some foods, the use of digital food photographs resulted in an estimation error at both the individual and group levels. Nevertheless, a digital photographic food atlas is a convenient portion size estimation aid, and the accuracy of estimates from food photographs could be improved through further modification of the portion sizes depicted in the food atlas. Moreover, future studies may consider combining food photographs with other portion size estimations (e.g. line diagrams and textual descriptions of portion sizes) that can flexibly estimate the portion sizes of complex dishes^(22,43,50)^. A previous study reported that amorphous foods and liquids were more accurately estimated using textual descriptions (i.e. estimating in grams or millilitres, standard portion sizes and household measures) than using photographs^(50)^. Thus, it may be better to provide several options for reporting portion sizes. Therefore, further development, refinement and testing of digital food photographs are needed to improve the accuracy of estimating the food portion size.

## Supplementary material

For supplementary material accompanying this paper visit https://doi.org/10.1017/jns.2022.102. click here to view supplementary material
