# Accuracy of estimates of serving size using digitally displayed food photographs among Japanese adults – CORRIGENDUM

**DOI:** 10.1017/jns.2022.113

**Published:** 2022-12-21

**Authors:** Nana Shinozaki, Kentaro Murakami

**Details of correction:** correction to wording of abstract

**Text reads:**

**“**At the study site, fifty-four participants aged 18–33 years were served fourteen foods in the amount they usually ate.”

**Corrected text should read:**

“At the study site, fifty-four participants aged 18–33 years served fourteen foods in the amount they usually ate.”

