# Supplementary material for: Accuracy of estimates of serving size using digitally displayed food photographs among Japanese adults
Source: J Nutr Sci. 2022 Nov 24;11:e105. doi: 10.1017/jns.2022.102 (PMC9705702; doi:10.1017/jns.2022.102)
Supplement: Supplementary file 1 [file jnssup.zip › S2048679022001021sup001.pdf]

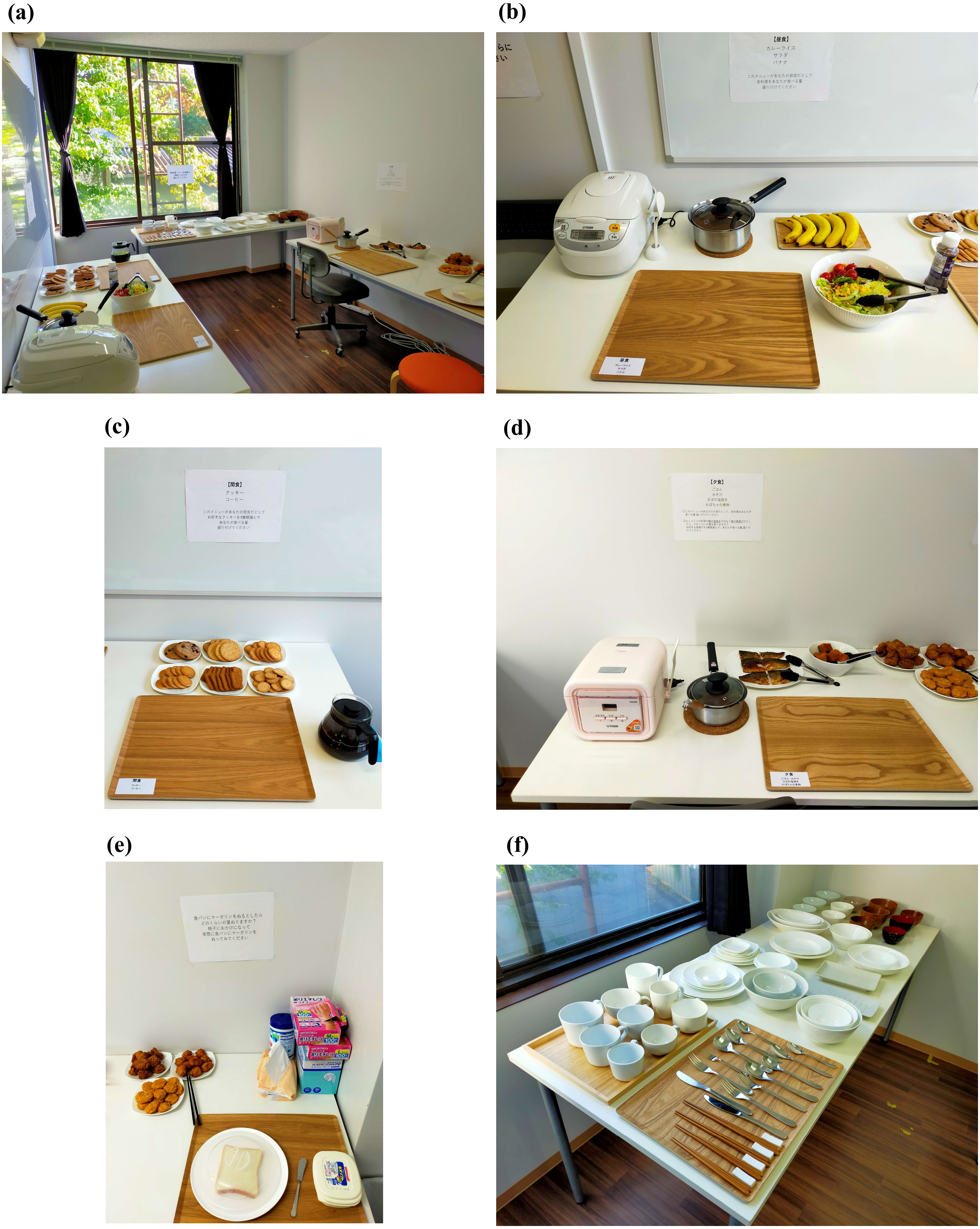

Supplemental Figure 1. Study site for serving sessions. (**a**) The view of the whole room. The places to select (**b**) lunch menu, (**c**) snack menu, (**d**) dinner menu, and (**e**) margarine. (**f**) The place to select tableware.
