# Supplementary material for: Accuracy of estimates of serving size using digitally displayed food photographs among Japanese adults
Source: J Nutr Sci. 2022 Nov 24;11:e105. doi: 10.1017/jns.2022.102 (PMC9705702; doi:10.1017/jns.2022.102)
Supplement: Supplementary file 1 [file jnssup.zip › S2048679022001021sup002.pdf]

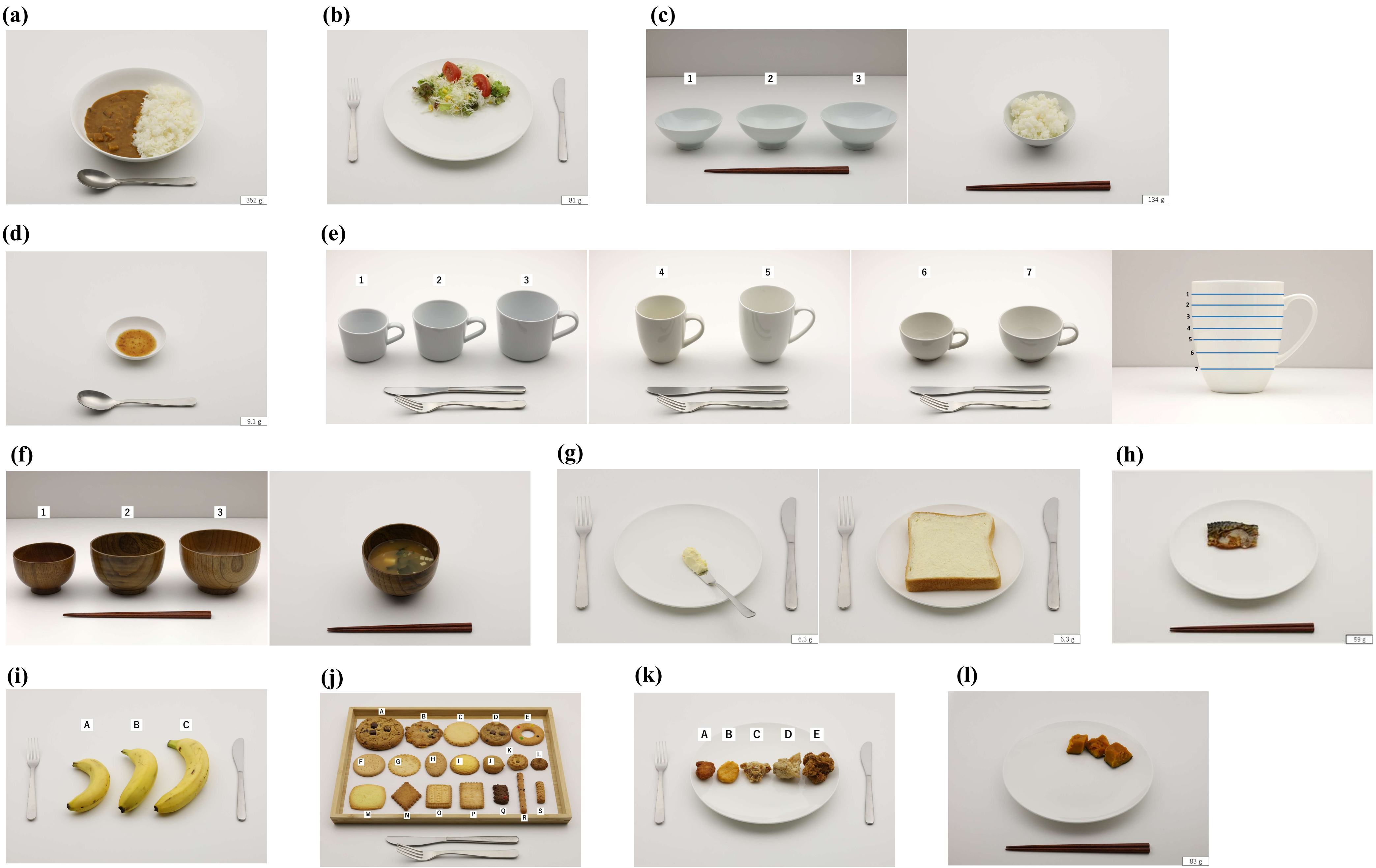

Supplemental Figure 2. Photographs used to estimate serving sizes. (**a**) curry and rice, (**b**) salad, (**c**) a bowl of rice, (**d**) dressing, (**e**) coffee, (**f**) miso soup, (**g**) margarine, (**h**) grilled mackerel, (**i**) banana, (**j**) cookies, (**k**) Japanese fried chicken, and (**l**) simmered squash. A picture of the fourth-smallest portion size is shown in the photos **a–d**, **f–h**, and **l**.
