# Supplementary material for: Accuracy of estimates of serving size using digitally displayed food photographs among Japanese adults
Source: J Nutr Sci. 2022 Nov 24;11:e105. doi: 10.1017/jns.2022.102 (PMC9705702; doi:10.1017/jns.2022.102)
Supplement: Supplementary file 1 [file jnssup.zip › S2048679022001021sup003.pdf]

(**a**) Curry and rice

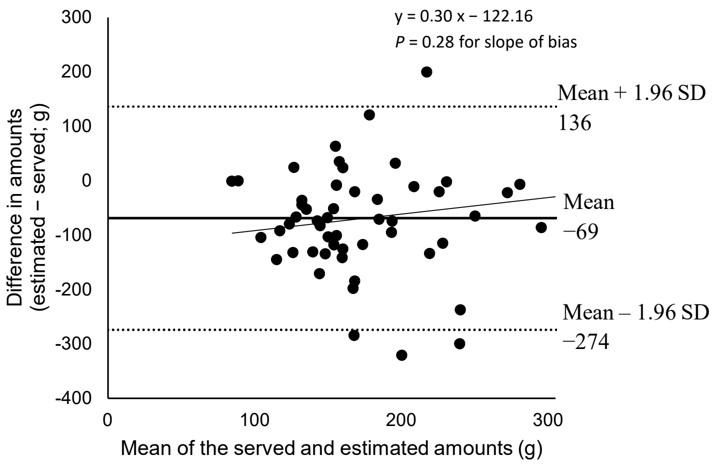

(**b**) White rice of curry and rice

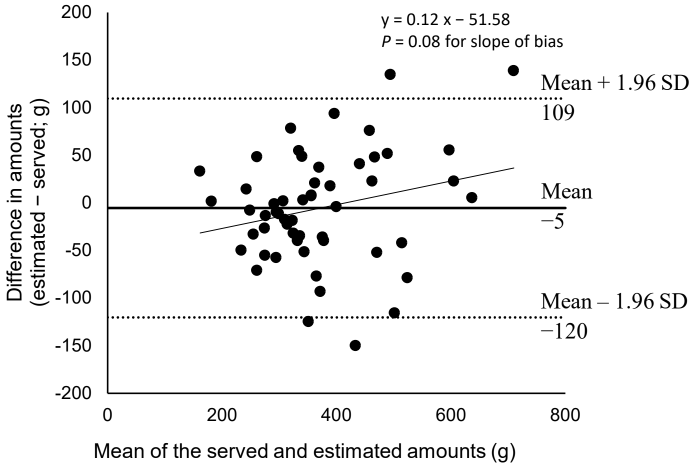

(**c**) Curry sauce of curry and rice

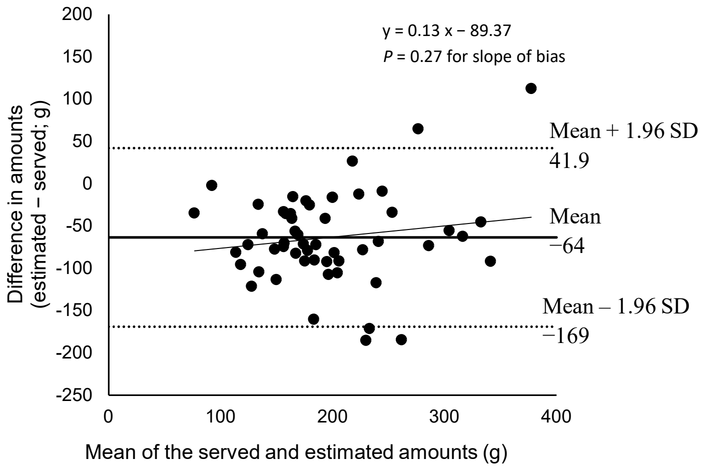

(**d**) Salad

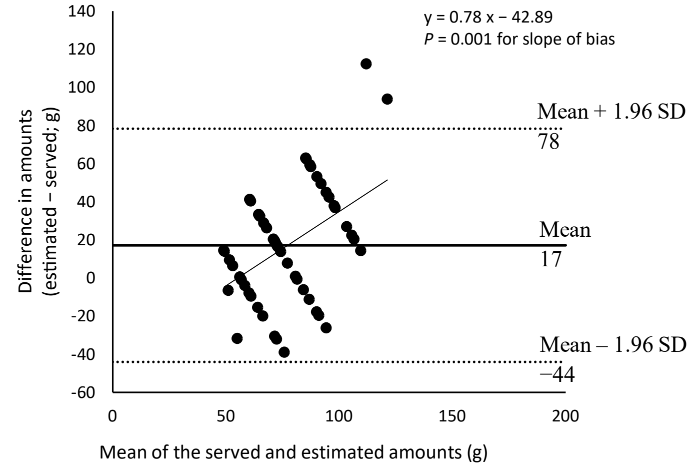

(**e**) Dressing

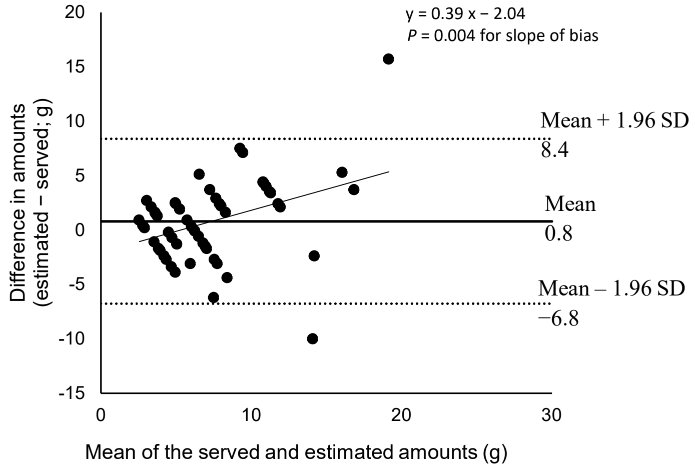

(**f**) Banana

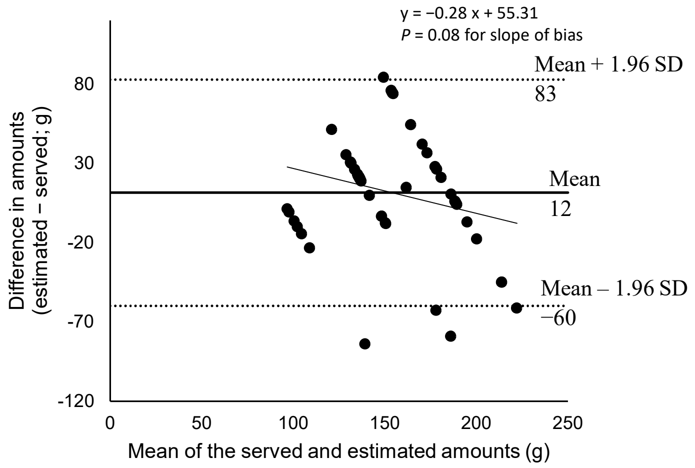

(continued)

Supplemental Figure 3. Bland–Altman plots assessing the agreement of the served and estimated food amounts in 54 Japanese adults: (**a**) curry and rice, (**b**) white rice of curry and rice, (**c**) curry sauce of curry and rice, (**d**) salad, (**e**) dressing, (**f**) banana, (**g**) cookies, (**h**) coffee, and (**i**) margarine. The solid line represents the mean difference, and the dotted line represents lower and upper limits of agreement with a solid regression line added. The *P*-value of the slope of bias was <0.05 for salad, dressing, coffee, Japanese fried chicken, and margarine.

(**g**) Cookie

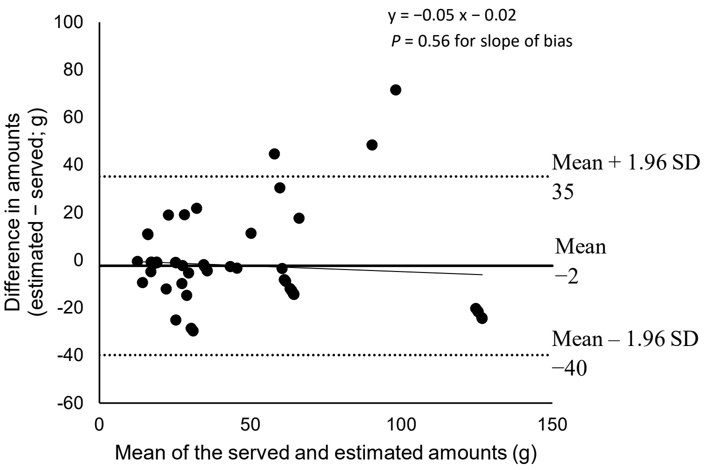

(**h**) Coffee

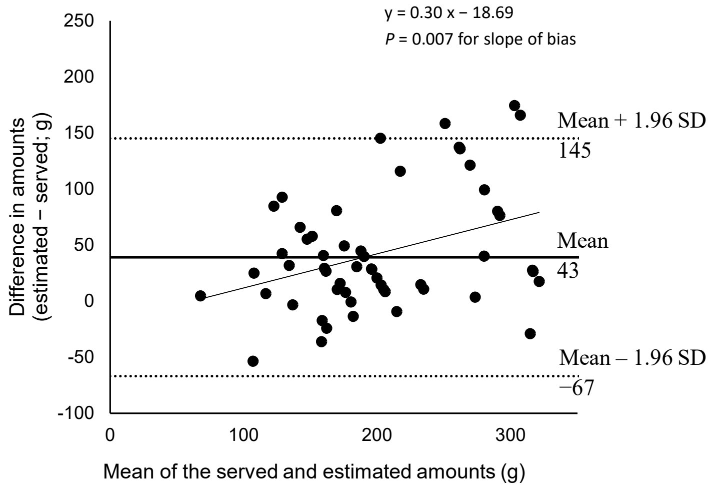

(**i**) Margarine

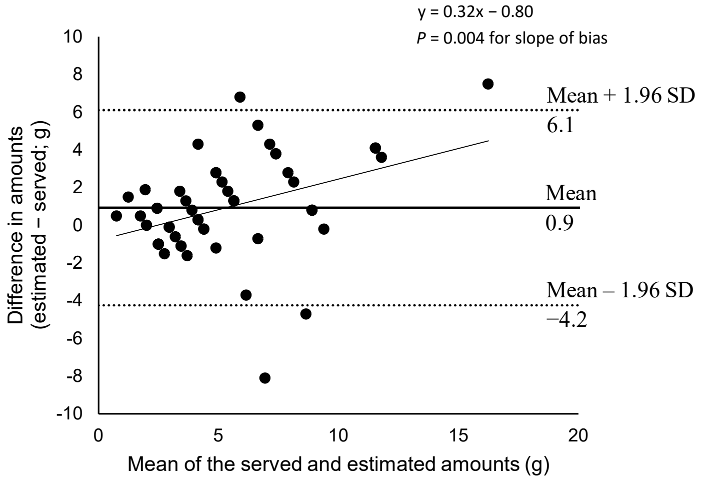

Supplemental Figure 3. *(continued)*
